# Transient Charge Collection in Ultra-Thin SiC Membranes for Single-Ion Detection

**DOI:** 10.3390/s26061809

**Published:** 2026-03-13

**Authors:** Enrico Sangregorio, Alfio Samuele Mancuso, Saverio De Luca, Annamaria Muoio, Lucia Calcagno, Francesco La Via

**Affiliations:** 1 Consiglio Nazionale delle Ricerche-Istituto per la Microelettronica e Microsistemi (CNR-IMM), VIII Strada n°5, 95121 Catania, Italy; alfio.mancuso@phd.unict.it (A.S.M.); saverio.deluca@cnr.it (S.D.L.); annamaria.muoio@imm.cnr.it (A.M.); francesco.lavia@cnr.it (F.L.V.); 2 Department of Physics and Astronomy, University of Catania, 95123 Catania, Italy; lucia.calcagno@unict.it

**Keywords:** silicon carbide detectors, TCAD simulations, charge collection dynamics, membrane sensor, deterministic ion implantation

## Abstract

Silicon carbide (SiC) detectors continue to emerge as a promising technology for applications requiring radiation hardness, fast response times, and stable operation in harsh environments. In this work, the charge-collection dynamics of ultra-thin membrane SiC detectors are investigated through time-dependent TCAD simulations, consistent with previously reported measurements. The study analyzes the transient response following the localized generation of electron–hole pairs induced by ions, comparing bulk and membrane detector geometries with identical active-layer thicknesses. Two-dimensional simulations provide a time-resolved characterization of the electron and hole current-density distributions within the active region of the device. The results show that both device architectures present a transient current signal featuring two main components. Despite similarities in the prompt drift-driven signal component, the SiC membrane response is characterized by a short secondary component returning to zero within 3.5 × 10^–10^ s at zero external bias, making it well-suited for reliable single-ion detection. In contrast, bulk devices exhibit a markedly different response, characterized by a significantly more intense and prolonged secondary component followed by a long tail that does not return to zero within the simulation time window for all investigated reverse biases. This tail is the result of the collection of carriers generated in the substrate that reach the depletion region through diffusion-driven processes. These findings contribute to the optimization of SiC-based solid-state detectors for quantum-technology device fabrication, demonstrating that the removal of the substrate drastically reduces the diffusion-dominated current component, thereby ensuring precise timing and minimal charge loss.

## 1. Introduction

Quantum systems in wide-bandgap semiconductors have emerged as promising platforms for applications in quantum computing and sensing [1,2]. The quantum properties of color centers in diamond, such as nitrogen-vacancy centers [3], and in silicon carbide, such as neutral divacancies ($V_{\mathrm{Si}}V_{C}^{0}$) with a spin-triplet (S = 1) ground state [4], and negatively charged silicon vacancies ($V_{\mathrm{Si}}^{-}$) characterized by a spin-quartet (S = 3/2) configuration [5], can be exploited for ultra-high-sensitivity quantum sensing [6]. Similarly, single-dopant systems, such as shallow donors or transition metal impurities in 4H-SiC, have shown promising quantum properties [7].

The two main approaches for developing quantum systems in semiconductors suffer from limitations in scalability and reliability. Deterministic single-ion implantation for the formation of single dopant systems, was performed via lithographic techniques based on scanning probes [8,9,10], or direct low-current ion implantation with real-time monitoring systems based on secondary-electron emission [11,12] or integrated structure output such as electron–hole pairs capture in PiN diodes [13,14] or drain current modulation in FET structures [14,15]. While successful in generating single-dopant quantum structures, these strategies are limited by low scalability or constraints on the sample architecture. On the other hand, color centers based on vacancies can be generated through electrons [4], ions [16], and neutrons [17] irradiation. While ensuring faster and more scalable processes, these structures rely on stochastic generation of the quantum systems offering poor spatial resolution and limited control over the precise positioning of the color centers. To address this challenge, we recently proposed an in-beam single-ion detection approach for deterministic ion implantation based on an ultra-thin 4H-SiC detector [18]. SiC-based radiation detectors have gained increasing attention as a robust alternative to conventional silicon devices, owing to their superior radiation hardness and stable operation in harsh environments [19,20,21]. Moreover, their high carrier mobility and detection fidelity [22,23], combined with advanced doping-selective electrochemical etching processes [24,25], enable the fabrication of sub-micrometric membrane detectors for high fidelity ion detection. The transition from bulk to membrane geometries is crucial for transmission-mode applications, as the removal of the substrate minimizes energy loss and preserves the ion’s trajectory. As a result, these membrane structures can be integrated into in-beam single-ion counting systems. This configuration provides real-time monitoring of the number of ions reaching the substrate, enabling precise control of both single-ion doping and ion-irradiation levels, ensuring reproducible creation of quantum systems in the substrate.

While recent studies have demonstrated the feasibility of single-ion counting using these 4H-SiC membranes [18], the underlying charge-collection physics in such confined geometries remains less explored than their bulk counterparts. In conventional bulk detectors, a substantial fraction of charge is generated within low-field or field-free regions of the substrate, where transport is dominated by diffusion. A portion of these charges can reach the space charge region, giving rise to the characteristic long-tail component in the collected-charge transient, influencing the measured Charge Collection Efficiency of the device. Due to the absence of the bulk, in membrane devices, this phenomenon is absent or negligible, giving rise to a sharper transient peak. To better understand this behavior, Technology Computer-Aided Design (TCAD) simulations provide a powerful framework for investigating the spatial and temporal dynamics of charge transport in semiconductor detectors, enabling controlled studies of electric-field distributions and transient current responses. Although TCAD has been widely applied to standard detectors, detailed time-resolved simulations of 4H-SiC membrane structures remain limited, and the influence of geometry on carrier-extraction efficiency and on the suppression of diffusion-driven components has not been analyzed.

In this work, a first comparison of the charge-collection dynamics in 4H-SiC Schottky membrane and bulk detectors is presented. The two devices share identical active-layer thicknesses and doping concentrations but differ in their bulk architectures, allowing the impact of device geometry on carrier transport to be studied.

## 2. Materials and Methods

**Figure 1:**
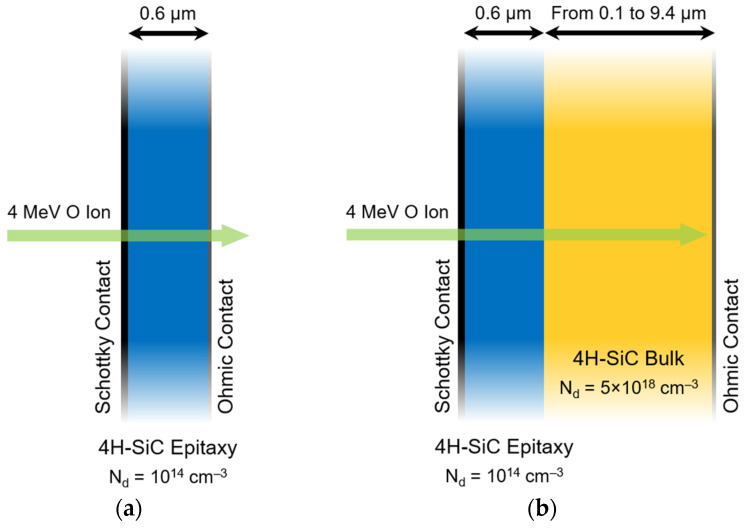
Schematic cross-sections of the (**a**) membrane and (**b**) bulk 4H-SiC devices used in the simulations. The green arrows indicate the ion-induced charge generation track.

The charge-collection dynamics of membrane and bulk 4H-SiC detectors were investigated through two-dimensional Technology Computer-Aided Design (TCAD) simulations performed with the Synopsys Sentaurus toolset [26]. Two-dimensional simulations were employed to reduce computational cost while still capturing the essential cross-sectional electric-field distribution and the dominant carrier-transport mechanisms of the devices. To investigate how the bulk substrate influences the collected charge transients, five structures were simulated: one membrane device and four bulk devices. Figure 1a,b schematically show the simulated geometries of the membrane and bulk devices, respectively. Each device featured a 0.6 µm thick epitaxial layer with a uniform n-type doping concentration of N_d_ = 1 × 10^14^ cm^−3^. The structures were modeled as Schottky barrier diodes, incorporating a top Schottky contact with a barrier height of 1.7 eV. For the membrane-based geometry (Figure 1a), the device was modeled as a free-standing epitaxial layer, in accordance with the selective etching-based fabrication method [24,25]. This approach involved omitting the substrate and positioning the back Ohmic contact directly on the rear surface of the simulated epitaxial structure. In contrast, the four bulk devices were modeled with increasing substrate thicknesses, specifically 0.1, 0.4, 4.4, and 9.4 µm (Figure 1b), using a substrate doping concentration of N_d_ = 5 × 10^18^ cm^−3^. For these structures, the Ohmic contact was placed at the bottom surface of the bulk layer. The active layer thickness and contact geometry were defined in accordance with the devices characterized by Sangregorio et al. [18].

Mesh refinement was applied in the active region, around the Schottky junction, at the epitaxy/bulk interface, and around the charge-generation area to ensure convergence and spatial accuracy. Mesh-independence tests were performed to confirm that the transient response did not depend on the discretization.

Carrier transport was predicted using the drift-diffusion mechanism with doping-dependent mobility, Fermi–Dirac statistics, Shockley–Read–Hall and Auger recombination, incomplete ionization, and carrier-velocity saturation models enabled. Impact-ionization models were also enabled, although the applied fields remained well below avalanche-multiplication threshold. Material parameters for 4H-SiC were taken from the Sentaurus device library. The 4H polytype was selected to maintain consistency with previously validated experimental setups [18] and due to its established industrial maturity. Radiation-induced displacement damage and the resulting formation of deep-level traps were not included in the model, as under the specific conditions of this study (MeV ion energy range and 600 nm membrane thickness), the interaction occurs primarily in the electronic stopping power regime, with negligible nuclear displacement within the active volume.

All ion-induced charge-collection simulations were performed using a two-step numerical procedure. First, a Quasi-Stationary Mode (QSM) bias ramp simulation was employed to establish the steady-state electric field distribution in the devices. Final reverse-bias voltages ranging from 0 to 50 V were investigated for all geometries, and the resulting solutions at each bias point were used as the initial state for the subsequent stage. Then, a Transient Mode (TM) was initiated to model the generation and collection of electron–hole pairs induced by the ion track at each fixed reverse voltage. Ion-induced energy deposition was emulated using a spatially localized carrier-generation profile, defined by a two-dimensional Gaussian distribution with a width of 50 nm. This profile was centered within the device, extending through the epitaxial layer and into the substrate. Generation was triggered at t_1_ = 2 × 10^−10^ s after the bias ramp to ensure a stable electric field before the transient onset. The total number of generated electron–hole pairs was calibrated to match the energy deposited by a 4 MeV O ion, consistent with the experimental setup in [18]. For this ion species and energy, SRIM [27] simulations showed a constant stopping power of 2.5 MeV/µm throughout the 4H-SiC epitaxial region. This deposited energy was converted into charge carriers assuming an average ionization energy of 7.8 eV per electron–hole pair [28], corresponding to a Linear Energy Transfer (LET) of 5 × 10^−2^ pC/µm, which was the value implemented in the simulations. The total simulation time was set to t_9_ = 1 × 10^−9^ s, sufficient to capture the fast drift-dominated component and a significant portion of the prolonged diffusion-driven tail typical of bulk devices. This simulation time ensures that the ionization-induced carriers do not reach the boundaries of the simulated domain, thereby preventing numerical artifacts and ensuring that the transient response is governed by the internal device physics rather than boundary conditions.

To ensure a consistent comparison between membrane and bulk geometries, this analysis focuses on the hole current collected at the Schottky contact. This approach mitigates artifacts arising from the reduced substrate thickness employed in the simulations. Specifically, in reverse-biased 4H-SiC detectors, holes are collected at the front contact; their dynamics are primarily governed by the properties of the epitaxial layer and the epi-substrate interface, making them an excellent probe for diffusion-mediated signals. Conversely, electron collection at the rear ohmic contact would be artificially biased by the proximity of the contact and the reduced thickness of the computationally optimized bulk. Consequently, focusing on the hole component provides a more reliable characterization of the diffusion-mediated tail. Furthermore, absolute Charge Collection Efficiency (CCE) was not adopted as a reference parameter in this work. This approach accounts for the limited reliability of standard 4H-SiC recombination models in predicting absolute charge values and addresses potential numerical artifacts arising from the spatial and temporal discretization of the ion-induced charge track. Instead, the study relies on a comparative analysis of current transients to highlight the physical influence of the bulk substrate.

## 3. Results and Discussions

**Figure 2:**
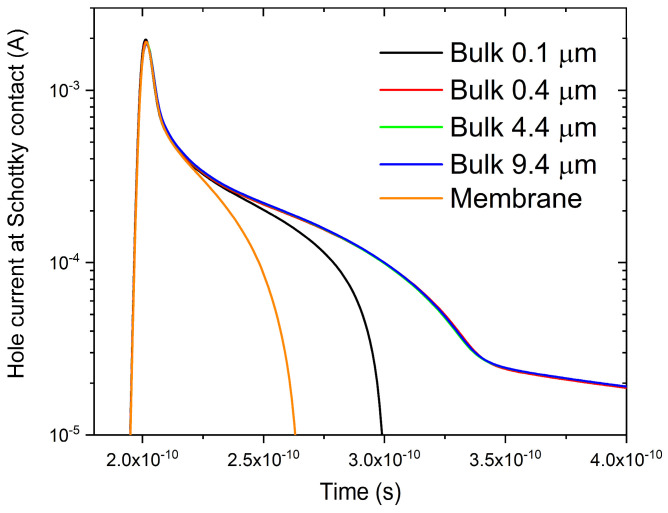
Transient hole current collected at the Schottky contact as a function of time for all simulated 4H-SiC detectors (membrane and different bulk thicknesses) under a reverse bias of V_R_ = 2 V.

Figure 2 shows the transient hole current collected at the Schottky contact as a function of time for all simulated bulk and membrane 4H-SiC detectors biased at a reverse voltage of V_R_ = 2 V. Following the electron–hole pairs generation event (t_1_ = 2 × 10^−10^ s), all simulated devices exhibit a sharp initial peak, corresponding to the rapid drift of carriers generated within the high-field portion of the epitaxial layer. The amplitude and temporal width of this component are comparable for all simulated structures, indicating a similar charge-generation and drift process in the epitaxial layer. The observed time scale of this initial response is consistent with the thin epitaxial active layer, where carriers generated within the depleted region are extracted within a few picoseconds. Interestingly, a secondary, slower component is observed in all structures, including the membrane detector. Here, this secondary component returns to zero within approximately 2.7 × 10^−10^ s, suggesting that even within the thin epitaxial layer, a fraction of the carriers is generated in low-field regions (e.g., near the back contact) where transport is slower. By contrast, the transient responses of the thicker bulk devices (0.4, 4.4, and 9.4 µm) are almost indistinguishable under the simulated conditions. This behavior is a consequence of the competition between carrier diffusion and recombination. Due to the high doping concentration of the bulk, only the minority carriers generated in close proximity to the epitaxial-substrate interface have a significant probability of reaching the depletion region. Consequently, the signal contribution from charges generated in deeper regions of the substrate becomes negligible. For this reason, the 4.4 µm configuration is selected as a representative case for thick bulk devices in the subsequent analyses. In these structures, the secondary component is significantly more prolonged than that of the membrane, persisting up to 3.3 × 10^−10^ s. More significantly, this component is followed by a low-intensity tail that does not return to zero within the simulated time window, consistent with the long-range diffusion of carriers from the deeper substrate regions. Finally, the 0.1 µm bulk device shows an intermediate behavior. It is the only bulk configuration where the signal returns to zero within the simulated timeframe. However, its decay dynamics remain distinct from the membrane case, with the secondary component returning to zero at approximately 3 × 10^−10^ s. This indicates that even a very thin substrate introduces a measurable change in the charge-collection dynamics, due to the contribution of carriers diffusing from the non-depleted regions.

**Figure 3:**
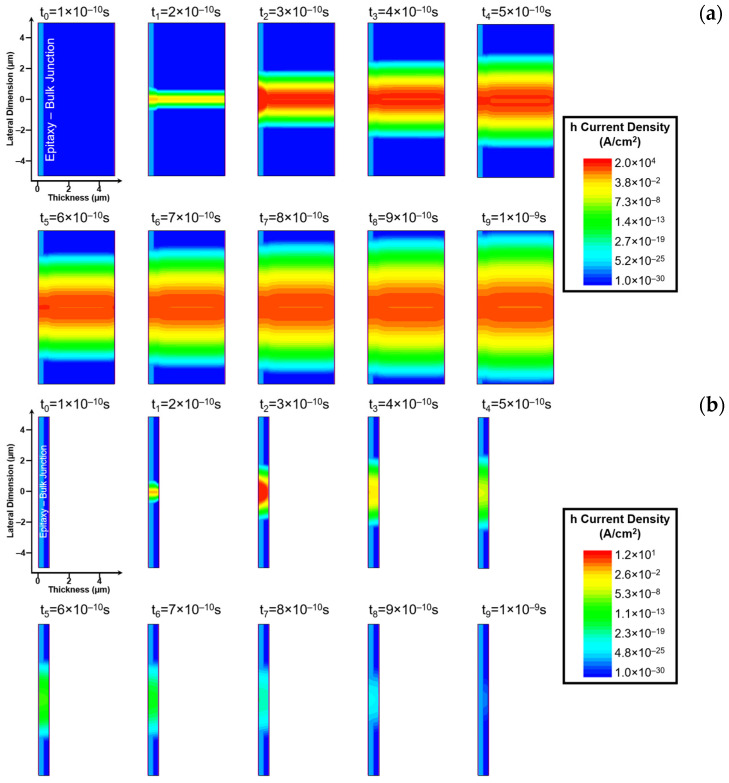
Time evolution of the hole current density obtained from TCAD simulations for 4H-SiC Bulk devices with (**a**) 4.4 μm substrate; and (**b**) 0.1 µm substrate. A slight contrast, particularly evident in the initial frames, allows for the visual differentiation between the epitaxial layer and the bulk substrate.

To better understand the origin of the long tail observed after the drift-dominated current peak, Figure 3a,b show the time evolution of the hole current density within the bulk detectors with a 4.4 μm and 0.1 μm substrate, respectively. The maps cover a temporal window starting from t_0_ = 1 × 10^−10^ s, representing the quasi-stationary state reached after the bias sweep, up to t_9_ = 1 × 10^−9^ s. The snapshots are equally spaced in time and capture the onset of electron–hole pair generation at t_1_ = 2 × 10^−10^ s. The comparison focuses on these two specific bulk geometries to highlight the impact of the substrate thickness on the persistence of the diffusion current. While the ultra-thin membrane exhibits an almost instantaneous carrier extraction with the current density dropping to zero shortly after the drift peak, the bulk structures allow for a direct visualization of the hole dynamics within the non-depleted region. In both devices, the frame at t_2_ = 3 × 10^−10^ s is temporally located after the drift peak observed in Figure 2, while the subsequent frames capture the hole current density during the long tail following the drift response. In the 4.4 µm bulk device (Figure 3a), the persistence of a long tail in the transient response is confirmed by the presence of non-negligible hole current densities distributed within the substrate throughout the entire simulated time window. Even after 1 ns, the hole current density remains non-zero, highlighting the slow contribution of diffusion-driven transport to the overall signal. In contrast, the 0.1 µm device (Figure 3b) exhibits rapid extraction of holes toward the Schottky contact, with minimal lateral spreading and no pronounced tail. The hole current density becomes negligible after t_4_ = 5 × 10^−10^ s, reaching values on the order of pA/cm^2^, reflecting the absence of a thick substrate and confirming the fast charge-collection dynamics of this geometry.

Notably, in all analyzed snapshots of Figure 3, the lateral expansion of the hole current density remains well within the simulation domain. This highlights that both the device dimensions and the simulation time window were appropriately chosen to prevent any interaction with the boundaries of the simulated structure, thereby avoiding edge effects that could alter the simulation results. The 2D maps in Figure 3 provide a spatially resolved picture of carrier transport, linking the temporal evolution of the transient current to the underlying distribution and motion of holes. This visualization highlights the significant impact of the bulk substrate on the overall device response, particularly in sustaining the signal tail.

**Figure 4:**
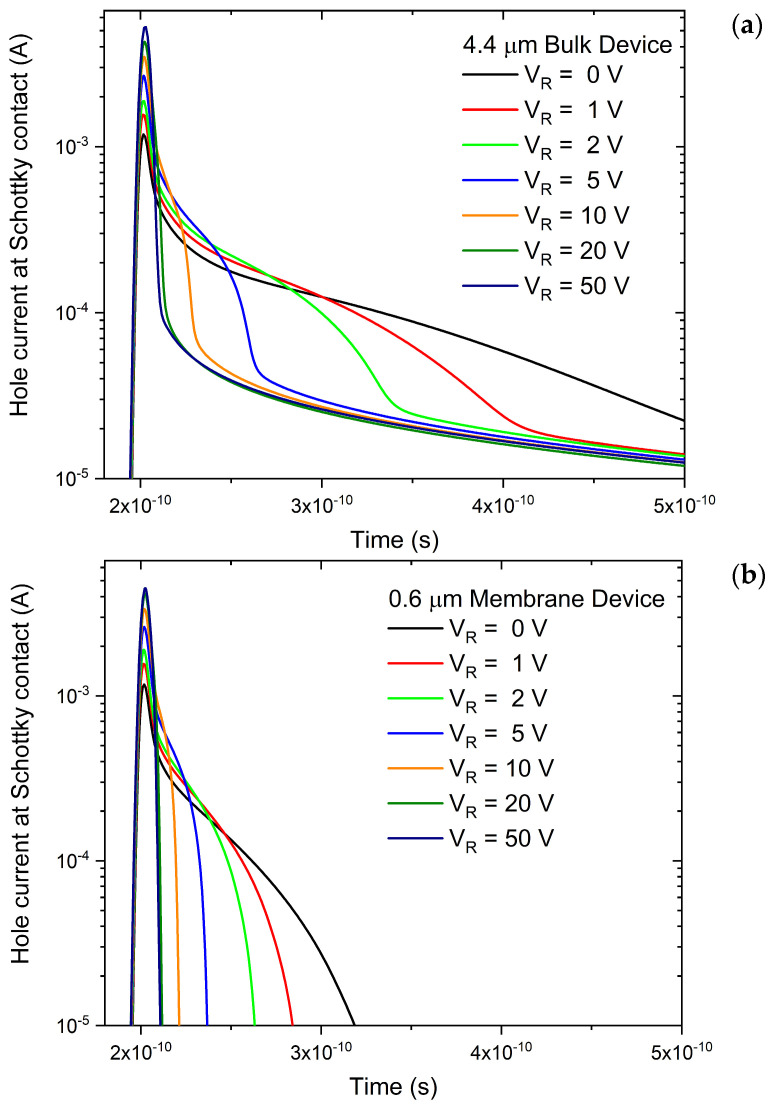
Transient hole current collected at the Schottky contact as a function of time for (**a**) the bulk 4H-SiC detector with a 4.4 μm substrate and (**b**) the membrane detector, under reverse bias voltages ranging from 0 to 50 V.

By applying a reverse bias, the electric field within the depleted region is increased, directly influencing the carrier drift velocity and, consequently, the transient response of the detector. To investigate the effect of the reverse bias on charge-collection dynamics, reverse voltages in the range from 0 to 50 V were applied to the devices prior to the localized generation of electron–hole pairs. Figure 4a shows the transient hole current collected at the Schottky contact for the bulk detector with a 4.4 μm substrate under different reverse-bias conditions. At low reverse bias, the transient response of the bulk device is characterized by a pronounced second component following the main drift peak. As discussed in the previous section, this component originates from carriers generated in the low-field or non-depleted regions of the substrate, which are collected through slower transport processes. With increasing reverse bias, this secondary component progressively shortens and partially overlaps with the main drift peak, reflecting the enhanced electric field and the widening of the space-charge region. At V_R_ = 20 V, this second component is fully integrated into the main peak, so that the distinction between the prompt drift contribution and the second component becomes less evident. Despite this evolution, for all investigated bias values the transient response of the bulk device does not return to zero within the 1 ns simulation window. This behavior indicates that a fraction of the charge generated in the substrate continues to contribute to the signal through slow diffusion-dominated processes, which are only weakly affected by further increases in the electric field within the depleted region. Another important effect of increasing reverse bias is the increase in peak current intensity of the drift component. This behavior is consistent with the higher carrier drift velocity under stronger electric fields, resulting in faster charge extraction and reduced carrier recombination during transport. The response of the membrane detector under different reverse bias conditions (Figure 4b) shares several similarities with the bulk devices. In particular, the drift peak intensity follows the same trend observed in the bulk structures, increasing with the applied bias and maintaining comparable absolute values across all investigated voltages (as previously seen in Figure 2). The secondary component also exhibits a bias dependence similar to that of the bulk devices. As V_R_ increases, this contribution becomes more intense and shorter, eventually merging into the primary drift peak at V_R_ = 20 V. Despite this qualitative similarity, the secondary component of the membrane response is systematically shorter and less intense than its bulk counterpart, decaying completely before 3.5 × 10^−10^ s, whereas in the bulk device this component is significantly longer. The late-time behavior represents the most significant distinction, as the membrane transient returns to zero for all bias voltages immediately after the decay of the secondary component. This is a direct consequence of the absence of a thick substrate, which effectively suppresses the diffusion-driven charge transport that contributes to the signal over extended time scales in bulk devices. By eliminating this contribution, the membrane response remains temporally confined, confirming that the substrate is the only source of the persistent tails observed in standard detectors.

## 4. Conclusions

In this work, the charge-collection dynamics of bulk and membrane 4H-SiC Schottky detectors were investigated through time-dependent TCAD simulations. By directly comparing devices with identical active-layer thickness and doping concentration but different substrate geometries, the influence of the bulk region on the transient response was studied. Transient current analysis demonstrates that both bulk and membrane detectors exhibit a fast, drift-dominated initial peak associated with carrier collection in the 0.6 µm epitaxial layer. This primary response occurs on sub-nanosecond time scales, consistent with the thin active region of the devices. Significant differences emerge in the subsequent stages of the signal. In bulk devices, the initial peak is followed by a prolonged secondary component and a persistent diffusion-driven tail originating from the substrate. This behavior saturates once a sufficiently thick substrate is simulated, showing no significant differences between the 4.4, and 9.4 µm bulk configurations. Furthermore, as the reverse bias increases, this secondary component shifts toward shorter times, eventually overlapping with the drift peak at V_R_ = 20 V. Conversely, the prolonged tail remains independent of the reverse bias and does not return to zero within the simulation time window. In contrast, ultra-thin membrane detectors, while presenting a secondary component with a behavior similar to bulk devices under increasing reverse voltage, exhibit rapid and complete charge extraction, with the signal returning to zero within 3.5 × 10^−10^ s at zero external bias. Overall, these results demonstrate that ultra-thin 4H-SiC membrane detectors provide fast transient responses with minimal diffusion-induced components. This behavior makes ultra-thin SiC membranes particularly advantageous for in-beam single-ion detection, as their fast signal termination and suppressed diffusion tail enable precise timing and unambiguous single-event identification.

## Data Availability

The original contributions presented in this study are included in the article. Further inquiries can be directed to the corresponding author.
